# Evidence and gaps in the literature on HIV/STI prevention interventions targeting migrants in receiving countries: a scoping review

**DOI:** 10.1080/16549716.2021.1962039

**Published:** 2021-08-18

**Authors:** Faustine Kyungu Nkulu-Kalengayi, Robert Jonzon, Charlotte Deogan, Anna-Karin Hurtig

**Affiliations:** a Department of Epidemiology and Global Health, Umeå University, Umeå, Sweden; b Department of Sexual Health and HIV Prevention, The Public Health Agency of Sweden, Stockholm, Sweden

**Keywords:** Sexually transmitted diseases/STDs, behavioural, biomedical and/or structural interventions, sexual and reproductive health and rights, combination prevention, population mobility, randomised controlled trials/RCT, trend statement

## Abstract

**Background:**

Evidence suggests that migration increases vulnerability to human immunodeficiency virus (HIV) and other sexually transmitted infections (STI). However, there is limited knowledge about what has been done or needs to be done to address migrants’ vulnerability in receiving countries.

**Objectives:**

A scoping review was carried out to map the existing literature in this field, describe its characteristics, identify gaps in knowledge and determine whether a Sexual and Reproductive Health and Rights (SRHR)-perspective was applied.

**Methods:**

We used the Arksey and O’Malley framework and the Joanna Briggs Institute guidelines for scoping reviews and subsequent enhancements proposed by other authors. We searched three databases and grey literature to identify relevant publications.

**Results:**

A total of 1,147 records were found across the three electronic databases and compiled. Of these, only 29 papers that met the inclusion criteria were included. The review shows that research in this field is dominated by studies from the USA that mostly include behavioural interventions for HIV and HBV prevention among migrants from Latin America and Asian countries, respectively. None of the interventions integrated an SRHR perspective. The intervention effects varied across studies and measured outcomes. The observed effects on knowledge, attitudes, perceptions, behavioural intentions and skills were largely positive, but reported effects on testing and sexual risk behaviours were inconsistent.

**Conclusions:**

There is a need for good quality research, particularly in parts of the world other than the USA that will address all STIs and specifically target the most vulnerable subgroups of migrants. Further research requires greater scope and depth, including the need to apply an SRHR perspective and incorporate biomedical and structural interventions to address the interacting causes of migrants’ vulnerability to HIV/STIs.

## Background

The current scope, impact and complexity of international migration has generated complex challenges including public health challenges for both host societies and the migrants themselves [1]. Current estimates suggest that there were approximately 272 million international migrants in 2019, an increase from 248 in 2015 and 173 million in 2000. Almost all countries in the world are involved as destinations, transit or origin [2]. However, there is no universally accepted definition of the term ‘migrant’. The term ‘migrant’ in this review refers to all persons who have moved to another country and stay there for at least 3 months, which is the limit for a tourist visa in most countries.

Migrant health status is a highly complex issue, and their health problems are often compounded by the migration process. Newly arrived migrants tend to have better health than the general population in their receiving and home countries. This phenomenon is known as the ‘healthy migrant effect’ or ‘healthy migrant paradox’ [3]. However, studies have shown that this phenomenon is outcome specific and related to individual socio-demographic characteristics, experiences faced throughout the migration process and time since immigration [3–5]. When it comes to infectious diseases including HIV/STIs, migrants are generally at an increased risk than the general population in their destination countries because of the experiences and circumstances surrounding the migration process including the socio-economic and political factors in both the home and receiving countries [6–8]. However, the relationship between migration, mobility and HIV/STIs has been described as complex and controversial and depending on the stage and prevalence of infections and conditions in country of origin, transit and destination [9].

Migration has been identified in some regions as an independent risk factor for human immunodeficiency virus (HIV) infection and a key driver of the pandemic, which remains a major global public health challenge [8]. For instance, in the most low prevalence countries, HIV affects migrants to a great extent, particularly those who come from high prevalence countries, who are often overrepresented among HIV infection cases that are being reported [8]. Similar patterns have been reported in the USA and Canada [10,11]. In the European Union (EU)/European Economic Area (EEA) member states, the proportion of migrants among new HIV diagnoses varies and can reach up to 70% in some countries [12]. Furthermore, the proportion of migrants among new HIV diagnoses being reported in high-income countries surpasses the percentage represented by foreign-born in the general population [13]. Even migrants from countries without high HIV prevalence are at an increased risk of HIV infection due to several social, economic and political factors throughout the migration process that create and increase vulnerability to HIV and other Sexually Transmitted Infections (STIs) in all migrants [8,12,14,15]. Thus, there is a need for effective targeted HIV/STIs prevention interventions in destination countries.

HIV/STI prevention interventions refer to all interventions or programmes aimed at preventing the acquisition, spread and consequences of HIV and/or other STI infections. These include different strategies aimed at preventing exposed individuals from becoming infected (primary prevention), stopping onward transmission (secondary prevention) and improving the quality of life of those affected (tertiary prevention) [16]. These strategies involve various types of interventions to reduce risky behaviours (behavioural interventions), prevent HIV/STI acquisition and transmission, e.g. condom, testing, antiretroviral and STI treatments, pre- and post-exposure prophylaxis (biomedical interventions) and/or address underlying social, economic, political or environmental factors that make some individuals or groups more vulnerable than others (structural interventions) [17].

The Joint United Nations programme on HIV/AIDS (UNAIDS) recommends a rights-based approach to HIV prevention programming in order to address the interacting causes of HIV risk and vulnerability and reduce new infections [18]. This approach is known as ‘combination HIV prevention’ and is defined as a rights-based approach that ‘relies on evidence informed, strategic and simultaneous use of complementary behavioural, biomedical and structural prevention strategies, which operate on different levels (e.g. individual, interpersonal, community, societal) to address the specific and diverse needs of key populations at risk of HIV infection’, including migrants [18]. This approach has its roots in the right to health as a fundamental human right and underlines the necessity to integrate sexual and reproductive health and rights (SRHR) in prevention efforts against HIV and other STIs [19]. The right to health further requires four key standard dimensions on healthcare services, i.e. availability (A), accessibility, acceptability (A) and quality (Q) also known as the AAAQ-framework. This framework has been formulated by the United Nations’ Committee on Economic, Social and Cultural Rights to articulate what the right to health meant in practice. The AAAQ concept stresses that healthcare services, including HIV/STI services should be available (in sufficient quantity), accessible (physically, economically including accessibility of information and without discrimination), acceptable (ethically and culturally appropriate, and sensitive to gender, age and diversity in the population) to all particularly the most vulnerable groups, and the services should be of good quality (medically and scientifically appropriate and of the highest quality) [20].

The World Health Organization (WHO) describes SRHR/HIV linkages as ‘concerted efforts in policy, programmes, and service delivery that support comprehensive sexual and reproductive health needs and rights of all people, including migrants, within a framework of gender equality and human rights’ [19]. SRHR encompasses the right of all individuals to make decisions concerning their sexual activity and reproduction free from discrimination, coercion and violence, and the right to a health system that provides the same opportunities (in terms of availability, accessibility, acceptability and quality) for all people to achieve the highest attainable health [21]. However, an SRHR perspective on HIV entails the recognition of the intrinsic connections between SRHR and HIV and the necessity of a bi-directional linkage between SRHR and HIV responses at the policy, systems and service delivery levels to achieve human rights, gender equality and the sustainable development goals [19].

Migrants are often identified as one of the ‘key populations’ for HIV prevention efforts within the global AIDS response [8,16]. Consequently, international, regional (e.g. EU) and national policies and guidelines for HIV prevention emphasise not only their increased risk and vulnerability but also the benefits of interventions targeting migrants for both the individual and the host society [8,22]. These policies and guidelines also highlight the necessity of integrating other STIs in the response to the HIV pandemic and all related preventative work because HIV and STIs are interconnected in many aspectsfor example, regarding transmission, behavioural factors and potential control measures [23,24]. However, migrants not only have special needs, but also face complex barriers in accessing available healthcare services in receiving countries, which exacerbates their risk and vulnerability [7,8]. These barriers include legal status, discriminatory policies, stigma and discrimination within migrant groups and among health professionals, culture and language, knowledge and attitudes towards HIV and health care, racism and xenophobia, and low socioeconomic status [12,15].

Poor access or even a lack of access to available services can result in late HIV diagnosis and treatment with increased morbidity and mortality, as well as an increased risk of onward transmission and thus a high risk of post-migration infection [12,15,25]. The evidence suggests that there is a high level of post-migration acquisition of HIV, even though most of the migrants living with HIV were believed to have contracted HIV prior to migration [16,26,27]. Moreover, the decline in AIDS cases generated by HIV ARV therapy that has been reported across the EU has not been observed among the vulnerable migrant subgroup [28]; this suggests a failure of both primary and secondary prevention and the need for tailored interventions. However, there is limited knowledge about what has been done and what needs to be done in the field of HIV/STI prevention and migration. Specifically, what kind of preventive interventions have been implemented so far, whether they have been successful or not and whether the right (SRHR) perspective has been applied or not.

## Aim, research question and objectives

The aim of this scoping review was to map the research done in this area, identify any existing gaps in knowledge and determine whether an SRHR-perspective was applied.

The following research question guided this review: What does the literature tell us about HIV/STI preventive interventions targeting migrants in the context of their receiving countries, its effects and the use of an SRHR perspective?

The specific objectives of this scoping review were to:

- identify relevant literature on HIV/STI prevention targeting migrants, both peer-reviewed,
- describe the characteristics of selected studies and interventions, and identify gaps,
- examine reported effects and underlying theories and determine whether an SRHR perspective has been taken into consideration.

## Methods

### Protocol

The review protocol was drafted using Arksey and O’Malley [29] and the Joanna Briggs Institute guidelines for scoping reviews [30] and subsequent enhancements proposed by other authors [31,32]. However, the PRISMA extension for scoping reviews checklist for reporting published in 2018 was used during manuscript writing [33].

The draft was revised by the authors who also discussed the research question, its purposes, the search strategy, the number of databases to be searched and the overall protocol. A Swedish version of the final protocol is available from the first author on request. This review is an update of an early report published in Swedish by the PHAS in 2018 that was not peer-reviewed [34].

### Information sources and search strategy

We used a three-step search strategy as suggested by Arksey and O’Malley to identify relevant documents and papers [29]. The first author conducted an initial search of a number of bibliographic databases and key websites from their inception until 15 August 2016 to identify appropriate search terms and appreciate the standard and volume of available literature on this topic. This preliminary search was performed with the assistance of a research librarian, who helped customize search and build search terms. Relevant key and Medical Sub-Heading (MeSH) terms were identified at this step including key MeSH terms such as ‘Transient’ which was often combined with the term ‘Migrant’ to identify the study population. In addition, ‘Sexual violence’ and ‘Gender-based violence’ which are considered as risk factors for HIV/STIs and indicators of human rights violations including SRHR were identified as key terms [8]. Even key terms related to the essential standards of health services with respect to SRHR [20,21] were also selected and used in subsequent searches. The databases searched were PubMed, Web of Science, Scopus, Cochrane Database of Systematic Reviews, PROSPERO (International Prospective Register of Systematic Reviews), the Cochrane Library, the Cumulative Index to Nursing and Allied Health Literature (CINAHL) and Web of Science. Additional sources involved key websites including the Public Health Agency of Sweden, World Health Organization, European Center for Disease Control, Centers for Disease Control and Prevention, and the Joint United Nations Programme on HIV/AIDS (UNAIDS). No existing scoping or systematic review on the topic was found after the preliminary search.

Thereafter, the Population, Intervention/Exposure, Comparison, Outcomes (PICO) and types (T) framework was used to define and systematise the literature search (See Table 1). A second search was then undertaken across all included databases after consultation with the research team. Three databases (PubMed, CINAHL and Web of Science) that retrieved high numbers of records were then selected for the main search, which took place in September 2016. The third step involved hand searches and checking the reference lists of relevant papers, and documents.

**Table 1.** Search structure based on PICO components with inclusion and exclusion criteria

PICO concept	Inclusion criteria	Exclusion criteria
Population: Does the study sample include at least 50% of migrants?	All persons aged 18 or more who have moved to a country other than their usual country of residence for a period of more than three months (>3 months) so that the recipient country becomes their new country of residence	Internal migrants (migration within a country’s national borders), tourists, guest workers and students temporarily staying in another country
Intervention: Is the paper about an intervention that aimed to prevent HIV and STI transmission and/or promote sexual health outcomes?	All HIV/STI prevention and sexual health promotion interventions targeting migrants in host countries.	HIV/STI interventions targeting the general population or other non-migrant at risk groups or where migrants make up less than 50% of participants or interventions with other goals
Comparison: Does the data analysis described in the paper include a comparison between intervention and comparison groups and/or before and after intervention?	Interventions where measurements are made before and after, with or without the use of a comparison or control group	Interventions without measurements before and after or without comparison with other groups
Outcomes: Is the paper about an intervention/study that includes any of the following outcomes?	HIV and STI incidence and prevalence, knowledge, attitudes, behaviour and practice, HIV and STI testing, unwanted pregnancies, abortion, contraceptive use, medical visits, condom use, unprotected sex, gender-based violence, condom use, stigma and discrimination, self-efficacy, acceptability, access, availability, adaptation, structural indicators, policy and practice	Outcomes that are not related to HIV and STI prevention or sexual and reproductive health and rights.
Types of study designs/literatures: Does the study design/literature include any of the following?	Both peer-reviewed and grey literature based on quantitative methods such as randomised controlled studies (RCTs), non-experimental studies (observational studies), quasi-experimental studies and meta-analyses, as well as qualitative studies describing and measuring outcomes of interest among migrants and their perceptions and experiences of health-promoting interventions and HIV/STI prevention in receiving countries.	Cross-sectional studies, case reports and series, ideas, editorials, opinions and pilot studies

The search strategy was not limited by study design, language or year. Upon completion, the searches from each database were documented and imported into a specific folder in the EndNote X7 software tool for managing bibliographies, citations and references and shared with other review team members. Thereafter, all duplicates were removed. Relevant documents and reports from key websites were also added to the folder as they were pertinent for the introduction and discussion.

A follow-up search of the three databases and grey literature sources was conducted in June 2019 after publication of the initial review as a non-peer reviewed report by the PHA in Swedish in 2018. The publication of the Swedish report took precedence over the peer-reviewed publication in accordance with the agreement [34]. This complementary search retrieved two systematic reviews and the reference lists of these reviews were also searched. Contrary to the current review, these two reviews were limited in terms of their type of interventions [35], target groups and contexts [36]. Appendix 1 shows an example of a complementary literature search in CINHAL database.

### Eligibility criteria

Using the PICO framework, we created a search structure and selection criteria that formed the basis of pre-specified eligibility/inclusion criteria for the review. Selection criteria were further refined through an iterative process during the course of the work, depending on concept definitions (see Table 1). We included peer-reviewed journal papers if they focused on HIV/STI, targeted migrants and were published in English before 19 June 2019. We excluded papers that were published after 19 June 2019 or did not fulfil the inclusion criteria. Table 1 displays the search structure with inclusion and exclusion criteria.

### Selection of sources of evidence

We used a two-stage selection process to assess the relevance of identified papers. First, the first author reviewed titles and abstracts to determine eligibility based on the deﬁned inclusion and exclusion criteria (see Table 1). At this level, consultations with other research team members guided decisions about any uncertainty with a title or abstract. The second level involved two independent reviewers who reviewed full texts using the eligibility criteria. At this level, when differences arose, the reviewers consulted with the other team members to reach a consensus.

### Data charting process and data items

Data extraction was performed by the first author and verified by the other team members and co-authors. The Transparent Reporting of Evaluations of behavioural and public health interventions with Nonrandomised Design (TREND) statement checklist was used to guide the extraction and charting of the data [37]. This statement consists of five main domains (title and abstract, methods, results and discussion) that include a checklist of 22 items on standard information about the publication (see S1 appendix). Additional information was added to identify the study source, authors, year of publication, country where the study was conducted, targeted issue and populations. Extracted data were compiled in two pre-tested standardised forms created in Microsoft Word after research team consultations. One table contained information about the study characteristics and the other described interventions in more details. The review team jointly reviewed all extracted data in a seminar. The charting forms can be found in an additional file. (See Appendix 2).

### Critical appraisal of individual sources of evidence

While scoping reviews do not generally include a quality assessment of studies, it is suggested that such an assessment could be valuable in improving rigor and interpretation [31]. We therefore used the TREND statement checklist to assess the quality of included studies. Each of the 22 items of the TREND checklist was assigned one point for a positive response and zero for a negative one. For multi-items that consisted of more than one question, each question was assigned the equivalent of 1/number of questions in the item. Thereafter, a total score for each paper was estimated by totalling all points for each individual item. The theoretical range was 0 to 22 points, with a higher score indicating ‘high quality’. The resulting scores were further collapsed as follows: papers that scored at least 17.6 (80%) out of 22 points were graded as a ‘high-quality study’, papers that scored less than 17.6 (79.7%) but at least 14.5 (66%) points were rated as a ‘medium quality study’ and those that scored less than 14.5 (66%) points were considered a ‘low-quality study’

### Synthesis of results

Due to the heterogeneity of the existing literature and the fact that grey literature mainly provided contextual information on the extent and reasons for the vulnerability of migrant populations to HIV/STIs, and existing prevention strategies and approaches, the synthesis for this review was limited to peer-reviewed published work. It consisted in a numerical summary (quantitative analysis, e.g. simple numerical counts) and a thematic analysis (qualitative analysis) of components of both studies and interventions in the included papers [38].

### Results

**Figure 1.**
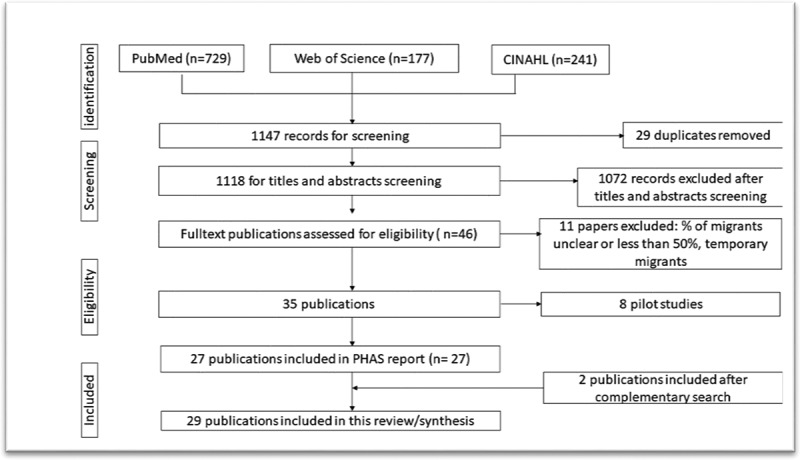
Flow chart of the literature search and selection process

A total of 1,147 records were retrieved, and after duplicates were removed, 1118 remained. After abstracts and full-text screenings 1092 papers were excluded, and 27 were included in the initial report [34]. Two more papers were added after the complementary search [39,40]. Figure 1 illustrates the literature search and selection process.

### Study characteristics

Most included papers (n = 26) reported single studies, but one paper contained two studies [41] and another overlap with a previously published paper [42,43]. The papers were predominantly (n = 16) from the USA of America (USA) [43–58] and published between 1993 and 2017. All studies were quantitative, except one in which a mixed methods design was used [59]. Half (n = 14) of the quantitative studies were randomised controlled trials (RCT) [42,43,45–48,52,54–56,58,60–62].

Most studies focused on HIV prevention (n = 16) followed by Hepatitis B Virus (HBV) prevention (n = 9) and mainly targeted migrants from Latin America [46–51,53,55–57,63] and Asian countries [40,42,43,45,52,54,58,61,62] respectively. The remaining focused on HIV/STI prevention among sub-Saharan African [64] and Caribbean [65] migrants, and sexual health promotion among migrants from Vietnam [59], sub-Sahara Africa, South East and Middle East Africa [39]. Most studies (n = 17) included both sexes/genders [39–43,45,48–50,52,54,58,61,62,64,66,67], seven focused exclusively on women [44,46,47,53,55,59,65] and five targeted only men [41,51,56,57,60]. The sample sizes ranged from 18 to 1500, with migrant shares varying from 55 to 100% across studies. The study populations in included papers were defined by migratory status alone [59,64] or in combination with country of birth [40,43,45–49,51,52,63], country of origin/citizenship [41,44,51,54–56,58,60–62,64,66,67], ethnic origin/race [39,42,43,46,47,50,51,53–56,65]. The concepts used to identify them included immigrants [56,60,66,67], migrants [40,41,59,63], first-generation migrants [65], refugees [64], related ethnicity/race [39,46,47,49–54,56], citizenship, birth or home country [44,45,48,58,60–62]. The oldest participant was 86 years old and the youngest was 13 years old. The majority (n = 20) of studies included at least three outcomes [39–41,44,46,47,49,51–53,55,57–60,63–66], and the most frequently measured outcome was knowledge (n = 22) [39–41,43,44,46–52,55,57,58,60–66]. Detailed characteristics of the included studies are described in Table 2.

**Table 2.** Description of the characteristics of included studies

Authors, year and place	Study design	Sample size (n): (intervention/control)	Gender/Sex: Male/Female (%)	Age (years)	Country of origin (%)	Length of stay (years)	Outcome measures	Study period/ follow-up time (retention rate)
Juon et al, 2016, USA	RCT	232 (124/108)	Mixed (43/57)	Mean: 48.8	China (32), Korea (34) and Vietnam (32),	1–41 (16.82 ± 10.52)	Self-reported Hepatitis B Vaccination (verified with the medical records)	23 months/at 7 months
Merchant et al, 2015, USA	RCT	150 (n = 50) and an ED (n = 50) and two community-based organizations (n = 50)	Mixed: (unclear)	27–47 years	The Dominican Republic (43), Puerto Rico (22), Guatemala (17), El Salvador (7), Mexico (5), Bolivia, Columbia, Ecuador, and Venezuela (6).	Median: 17 (video) vs. 14 years (orally delivered information)	Comprehension of information (HIV knowledge Score)	7 months/ immediately after
Bastani et al, 2015, USA	RCT	1,123 (543/580)	Mixed: 35/65	18–64 (mean = 46)	Korea (97)	Average >16 years	HBV testing(self-reported)	2006–2012/at 6 months
Juon et al, 2014, USA	RCT	877 (441/436)	Mixed: 41/59	≥18	USA (2.7) Chine (35), Vietnam (32) and Korea (34)	0–54 (14.97 ± 9.83)	HBV screening (Self-reported)	7 months (November 2009 -June 2010/after 6 months
Takahashi et al, 2013, USA	RCT	200 (110/99)	Women:-/100	20–74	China (100)	Unclear	Knowledge about HIV risk factors, Knowledge about prevention and condom use skills	November 2010 to July 2012/at 3-months
Juon et al, 2013, USA	RCT	877 (441/436)	Mixed: 41/59		USA (2.7), China (35), Korea (34), Vietnam (32)	0–54 (14.97 ± 9.83)	HBV Knowledge	November 2009 and June 2010/ at 6 months
Chen et al, 2013, USA	RCT	260 (130/130)	Mixed: 40/60	16–74	Laos (73.1) Thailand (21.5)	≤ 10 years (37.9%), >10 years (62.1%)	HBV Knowledge, HBV testing and factors affecting testing	/at 6 months
Peragallo et al, 2012, USA	RCT	548 (274/274)	Women	18–50	Colombia (34), Cuba (13) in Cuba, Peru (8), USA (8), the Dominican Republic (6), and other 11 countries (5)	Unclear	Chlamydia incidence, condom use, get drunk, IPV, partner communication, perceived HIV risk, self-efficacy, HIV knowledge, norms, perceived barriers, intention to use condom, community prevention and depression	January 2008-April 2010/at 3-,6- and 12-months

Authors, year and place	Study design	Sample size (n): (intervention/control)	Sex/Gender: Male/Female (%)	Age (years)	Country of origin (%)	Length of stay (years)	Outcome measures	Study period/ follow-up time (retention rate)
Rhodes et al. 2011, USA	RCT	142 (72/70)	Men	18–53	Mexico (59) El Salvador (6.8), Guatemala (13.4) Honduras (9.9), Nicaragua (1), Other (7.7)	Unclear	Condom use, HIV testing	Unclear /at 3 months
Wingood et al 20,011, USA	RCT	252 (125/127)	Women	30.3 (± 6.86)	South America (38.2) Cuba (25.5), Central American (19.9), Dominican Republic (4), Puerto Rico (2), Mexico (1.6), USA (8.8)	9.2 (± 8.6)	Consistent condom use, Cultural norms: traditional views of gender roles, HIV knowledge, Perceived barriers to condom use, Self-efficacy for negotiating safer sex, Feelings of power in relationships, Condom use self-efficacy	August 2007-August 2010/ at 3- and 6-month
Taylor et al, 2009a, USA and Canada	RCT	460 (unclear)	Mixed (unclear)	≥ 50% were < 45 years	China (100)	Unclear	HBV testing (primary), HBV Knowledge (secondary)	Unclear (2005)/ Unclear
Taylor et al 2009b, Canada	RCT	325 (141/157)	Mixed (30/70)	*>18*	China (76) or other country (13)	< 2 years (146), ≥ 2 years (162)	Knowledge about HBV	2006–2007/at 6-month
Peragallo et al, 2005, USA	RCT	657 (404/253)	Women	18–44	Mexico = 89.4%, Puerto Rica (10.6)	1–21	HIV knowledge, Partner communication, Risk-reduction behavior intentions, Safer sex peer norms, Condom use and Perceived barriers to condom use	February 1999- October 2000/ at 3-and 6 months
McPhee et al, 2003, USA	RCT (*Posttest-Only* design with equivalent comparison group)	1500 (500/500/500)	Mixed	18–79	Vietnam (100)	24–46	Parents’ awareness, knowledge about HVB, Children vaccination	April 1998-March 2000/at 2 years
Kocken et al, 2001, Netherlands	RCT, without concurrent controls/ historical	589 (293/296)	Men	18–40	Turkish Morocco	<3 (n = 70), >3 (n = 494)	Misunderstandings about HIV, Risk appraisal for HIV infection, Perceived benefits of condom use, Perceived barriers for condom use, Condom self-efficacy and Intention to use condom	Unclear/
Roberts et al, 2017, Australia	Pretest-posttest design (single group)	18 (N/A)	Mixed (30/70)	14–21	Sub-Saharan Africa, South-East Asia and Middle East (unclear)	Unclear	knowledge, confidence, attitudes and skills in relation to sexual health	12 weeks
Rios-Ellis et al, 2015, USA	Pretest-posttest design	579 (N/A)	Mixed (43/57)	25–44	Foreign born (55), mainly from Mexico (97)	14.8 ± 11.8	HIV knowledge, Stigma, Risk perception and Willingness to discuss sexual risk with partner (communication)	12 weeks

### Critical appraisal within sources of evidence

Only one [45] of the 29 studies achieved a score that met the criteria of a high-quality study. Ten of the studies met the medium quality criteria [42,43,46–48,54,56,58,62,67] and the remaining 18 were graded as low-quality studies [39–41,44,49–53,55,57,59–61,63–66].

### Intervention characteristics

The interventions implemented in the included studies were predominantly behavioural (n = 25), either exclusively [39,41,43,44,46,48–50,55,59–61,63–66] or integrated with biological interventions [40,47,51–53,56–58,62]. Only four were exclusively biomedical [42,45,54,67].

Most of the 25 behavioural interventions largely involved exposure to prevention messages, education programmes through brochures, pamphlets, workshops, lectures, videos, media campaigns, theatre and drama to improve knowledge about HIV [41,44,46–50,55,57,60,63,65,66], HBV [40,43,52,58,61,62], HIV/STIs [47,53,55,64], sexual health [39], where to get tested [51] or receive condoms [44], and the ability to negotiate safe sex [47,53,55,59,65], communicate with one’s partner [47,49,53,55,65] and the community about HIV [66] or sexual health [39]. Behavioural interventions further aimed to address risky behaviours [41,47,49–51,53,55,57,59,60,63,65] and issues related to power in relationships [46,65], condom use promotion [41,44,46,47,53,55–57,59,60,64,66], HIV related stigma [49], social support [47] and internalised homophobia [57] as well as social norms [41,55,65] and gender roles [46] or mental [47] and sexual [65] health promotion.

The nine interventions that integrated behavioural and biological components [40,47,51–53,56–58,62] aimed to promote and increase the uptake of HIV [51,53,56,57] and HBV [40,52,62] testing and the HBV vaccine [58] or decrease chlamydia incidence [47]. The four interventions that only evaluated biomedical outcomes aimed to increase the uptake of the HBV vaccine [54] and testing [42,45,54] or monitoring pregnancy occurrence in HIV positive couples as a proxy of unprotected sex [67].

Sixteen of the interventions were theory-based [41,42,44–47,49,51–53,55–57,59,60,65]. The most commonly used theory was the social cognitive theory (n = 6), either alone [47,55] or combined with theories of gender and power [44,46,53], empowerment theory [56] or other theories [53]. The theoretical basis was unclear in 13 studies [39,40,43,48,50,54,58,61–64,66,67]. All theories used in the included studies are displayed in Table 4. None of the studies integrated an SRHR perspective.

The interventions were culturally and linguistically adapted to the target populations and mainly delivered at group level (n = 21) [41–50,53,55–57,59–61,63–66] as outreach activities by lay health advisors/peer educators [39,41,44–46,50,52–54,56,58,60,62–65]. Nevertheless, two of the interventions consisted of media campaigns alone [51] or combined with other delivery modes [58]. The settings varied across studies and consisted of different community venues [39,40,42,43,45,47,49–51,53,56–58,60,64], migrant shelters [41,63], participants’ homes [50,52,54,56,62,65], healthcare settings [44,46,48,53,59,67] and language schools [61]. The setting was not specified for one intervention [66]. Eleven of the interventions included a single session [41–43,45,48–50,60,61,63,67,68]; other interventions included two [44], three [54,61], four [46] or multiple sessions [39,40,47,51,53,56–58,65]. The number was not specified in three of the interventions [59,61,64]. The duration of sessions ranged from 10 minutes to 2 years. Incentives were provided for attendance in more than half (n = 16) of the interventions [43,44,46,47,49,51–54,56–58,61,62,64,65]. Other intervention details are illustrated in Table 3.

**Table 3.** Summary of key features of interventions

Authors and year	Theory	Target group	Setting	Deliverer	Unit and mode of delivery	Number and duration of session	Incentive	Language
RCT								
Juon et al, 2016	None	Asian Americans	Home based (outreach)	Lay health advisor	Individual/ Mails and phone calls	Three phone calls at month 1, 2 and 5 in 7 months	Yes	English, Chinese, Korean and Vietnamese
Merchant et al, 2016	None	Spanish-speaking Latinos	Medicine clinic, Emergency department and CBOs	Research assistant/ HIV counsellor	Group/Video and orally delivered	One 10 min (Orally delivered), one 15 min (Video)	Unclear	Spanish
Bastani et al, 2015	Health behavior framework	Koreans	Churches	Lay health workers	Group/ small group discussion and print materials	Single session	Unclear	Korean
Juon et al, 2014	PRECEDE–PROCEED planning model	Chinese, Koreans, Vietnamese	CBOs (churches, temples and schools)	Trained bilingual staff	Group/ information, role play video at the clinic and photo novel	One 30 min session	Unclear	Korean, Chinese and Vietnamese
Chen et al, 2013	Health Behavior Framework (HBF)	Hmong residents in the USA	Homes	Lay health workers	Individual (educational sessions and navigation services through home visits)	One 45 min	Yes	Hmong/ English
Juon et al, 2013	None	Chinese massage parlor women	CBOs (churches, temples, and schools)	Trained bilingual educator	Group (2–8)/educational session	One 30 min session/group over 1–2 weeks	Yes	Mandarin and Cantonese
Takahashi et al, 2013	Social Cognitive Theory and theories of gender and power	Chinese massage parlor worker women in the USA	Health center	Chinese female facilitators	Group (educational session and distribution of safe sex kits and information about free anonymous HIV testing)	Two 3 (intervention), one 2 h (control)	Yes	Mandarin/ Cantonese
Peragallo et al, 2012	Social cognitive theory of behavior change	Hispanic women	Accessible Community sites	Bilingual and bicultural Hispanic women (bachelor to doctoral degree)	Group (3–7)/role play, participatory sessions, videos and discussions	Five 2 hours sessions	Yes	Spanish/ English
Rhodes et al, 2011	Social cognitive theory and empowerment education	Heterosexual Latino men	**T**he ofﬁces of CBPR partners and in the homes of participants	Peer Educators (*companeros de salud*)	Group/ small group activities and discussions,, didactic teaching, DVD, role plays	Multiple sessions for HIV and one session for cancer program	Yes	Spanish
Authors and year	Theory	Target group	Setting	Deliverer	Unit and mode of delivery	Number and duration of session	Incentive	Language
Wingood et al, 2011	Social cognitive theory and theory of gender and power	Latina women	County HIV/AIDS office	Latina health educators	Group (7–8)/group discussions, role paying and teaching activities	Four 2.5 hours sessions during 4 consecutive weeks	Yes	Spanish
Taylor et al, 2009a	None	Chinese	Participants’ Homes	Lay health workers (Bicultural, trilingual Chinese Americans/Canadians)	Individual/ video and pamphlets through home visits	Unclear	Yes	Mandarin/ Cantonese
Taylor et al, 2009b	None	Chinese immigrant ESL students in Canada	CBO ESL schools	ESL teachers	Group (class sessions)	Three hours session	Yes	Mandarin/ Cantonese
Peragallo et al, 2005	Social cognitive theory of behavior change	Low-income Latino women in USA	Unclear	Bicultural staff and HIV counselor	Group (hands on activities, role playing, skill demonstration and homework)	Unclear	Unclear	Spanish/ English
McPhee et al, 2003	None	Vietnamese American parents	CBOs	Media, community members and staff	Community/radio spots, oral presentation, and broadcast announcements	Over 2 years	Yes	Vietnamese
Kocken et al, 2001	Health belief model	Turkish and Moroccan men in the Netherlands	Coffee houses and mosques	Peer educator	Group (lectures and discussions)	One, 75 min	Unclear	Arabic
NRCT								
Roberts et al, 2017	None		Migrant Resource Centre	Staff and peer educator		5 or more sessions/week		
Rios-Ellis et al, 2015	Airhihenbuwa and Webster’s PEN-3 model and Elder et al.’s framework	Underserved Latino in the USA	Community venues	Peer HIV positive and their relatives	Group (interactive group education and brochures)	One, 60–90 min	Yes	Spanish/ English
Galvan at al, 2015	None	Migrant and Returnee men in Mexico	Migrant shelters	Lecturer, peer, pamphlets	Group (group lecture, peer education and pamphlet	One 30 min (peer), one 5 h lecture	Unclear	Spanish
Veldhuijzen et al, 2012	None	Chinese migrants	Community venues	Staff/community members	Community campaign	12 months		
Drummond et al 2011	None	West African Refugees in Australia	Community hall	Peer educators	Group (workshops)	Unclear	Yes	English
Vega et al, 2011	Social identity theory	Latino gay men in the USA	CBOs	Bilingual program staff	Group (exposition and discussion)	5 sessions of unclear duration in 5 weeks	Yes	Spanish/ English
Martinez-Donate et al, 2010	Social-ecological framework and social marketing principles	Latino MSM who identify themselves as heterosexual in the USA	Community venues	Media	Community (mixed campaign)	7 months	Yes	Spanish
Authors and year	Theory	Target group	Setting	Deliverer	Unit and mode of delivery	Number and duration of session	Incentive	Language
Bertens et al, 2008	Problem-based learning (PBL), Trans theoretical Model (TTM), Self-regulated learning and observational learning	Afro-Caribbean women in the Netherlands	Homes	Peer health educators	Group (Group interaction and discussion)	Five sessions, unclear duration	Yes	Antillean/ Surinamese
Martin et al, 2005	None	Latino Migrants in the USA	Homes and Community venues	Peer community health workers	Individual/ Group (education session)	One session, unclear	Unclear	Spanish
Martijn et al, 2004	Theory of planned behavior	Arab speaking migrants in the Netherlands	Refugee shelters	Lay health worker and Professional Health advisor	Group (group lectures and discussions)	One session, 150 min	Unclear	Turkish/ Arabic
Busza et al, 2004	Community mobilization	Vietnamese illegal migrants sex workers in Cambodia	MSF clinics and drop-in centers	MSF Clinic staff	Group (participatory education, workshops, skills building)	Unclear	Unclear	Vietnamese
Kapplan et al, 2002	None	HIV positive Ethiopian and their sexual partners.	Regional HIV center	Ethiopian Cultural mediators/case Managers (CMs)	Individual or couple/ Counselling	One session, 150 min	Unclear	Amharic
Raj et al,2001	Social cognitive theory, empowerment model based on Freirian concept, self-in-relation, diffusion and innovation and theory of gender and power (Intervention 1), theory of reasoned action and health belief model (Intervention 2)	Hispanic women in the USA	Community center and community clinic	Community health worker	Group (education sessions)	12 sessions, 90 min, 12 weeks	Yes	Spanish
Shtarkshall at al, 1993	None	Ethiopian migrants in Israel	Unclear	Authors	Group (education session)	3 days	Unclear	Hebrew

### Intervention effects

#### Effect on knowledge, attitudes and perceptions

The interventions showed a positive effect on knowledge about sexual health [39], HIV/STIs [64], HIV [41,44,46–49,55,57,60,63,65,66], HBV [43,52,58,61,62], HVC and HIV testing services/locations [57]. However, the positive effect observed in the intervention group at the 6-month mark in Peragallo et al.’s (2012) study was not maintained at the 12-month follow-up [47]. A positive effect was also reported on attitudes towards sexual health [39], condom use [41,66] and HIV related stigma [49,66].

Reported effects on HIV risk perception [51,60,65], as well as the perceived ability to communicate with the community about HIV [59,66] or deliver safe sex messages to friends [39] were generally positive, except in Peragallo et al. (2012) in which no effect was demonstrated on perceived risk [47]. Positive effects were also observed on connectedness [57] and perceptions about cultural/traditional norms [46,65]. Despite this, Peragallo et al.’s (2005) early study detected no effect on safer sex peer norms [55].

#### Risk reduction and sexual risk behaviours and intentions

Reported effects on risk reduction and sexual risk behaviour outcomes varied across studies and outcomes. Despite reporting a positive effect on risk reduction behaviour intention in a previous study [55], Peragallo et al. (2012) identified no effect on self-efficacy for HIV prevention in a subsequent study [47]. Other studies showed positive effects on perceived behaviour control [41], intention [65], skills [59], and self-efficacy to negotiate safer sex [46,53] and communicate with partner [47,55,65]. Likewise, positive effects were observed on feelings of power in relationships [46], self-esteem [57], sexual assertiveness [65], as well as the intention to use condoms [47,53]. However, the positive effect on the intention to use condoms observed in Peragallo et al. (2012) at 6 months was not maintained at 12 months [47]. Positive effects were also reported on safer sex outcomes such as condom use [46,47,53,56,59], as well as related skills [44] and self-efficacy [46,60].

The reported effects on outcomes related to sexual risk behaviours were conflicting. While Peragallo et al. (2012) and Vega et al. (2011) demonstrated positive effects on intimate partner violence (IPV), drunkenness [47] and the number and type of sexual partners [57], respectively, Martinez-Donate et al. (2010) recognised contrasting effects on the number of incidences of unprotected sex with female partners between bisexual and heterosexual men [51]. However, the number of unprotected female sexual partners decreased in both groups, as well as the number of incidences of unprotected anal sex with male partners and unprotected male sexual partners among bisexual men during the post campaign [51].

#### Effect on testing and vaccination behaviours and intentions

The reported effects on HIV and HBV testing behaviours and intentions were also inconsistent across studies. Some studies reported positive effects [42,45,52,56], while others showed limited/no changes [49,53,62] or even negative effects [51] after the interventions. Positive effects were also observed on HBV vaccination behaviours [54], chlamydia [47] and pregnancy [67] incidence/occurrence.

### Discussion

This scoping review shows that research in this field is dominated by studies from the USA [43–58] that mostly include behavioural interventions for HIV and HBV prevention among migrants from Latin America [46–51,53,55–57,63] and Asian countries [40,42,43,45,52,54,58,61,62], respectively. None of the interventions integrated a SRHR perspective. The intervention effects varied across studies and measured outcomes. The observed effects on knowledge, attitudes, perceptions, behavioural intentions and skills were largely positive, but reported effects on testing and sexual risk behaviours were inconsistent.

### Lack of studies outside the USA

The review has revealed a scarcity of studies from outside the USA, including Europe. This might be an indication that HIV/STI prevention programmes targeting migrants in Europe are not systematically evaluated or published in peer re-viewed journals. This emphasises the need for interdisciplinary teams including scientists, policy makers and representatives from migrant groups with clear roles for designing, implementing and evaluating interventions and disseminating its findings. Another possible explanation is that the main research focus in this field might be on observational studies that describe prevalence, experiences, perceptions, needs and risk factors for HIV/STI among migrants rather than intervention studies that evaluate prevention programmes. Diaz et al. (2017) argued in their scoping review on interventions to improve migrant health that the paucity of studies in this field might be a consequence of the challenges faced in undertaking such research or the lack of priority of immigrants’ health in research policies [69].

### Vulnerable subgroups (of migrants) and other STIs not specifically targeted

The literature review also reveals that most studies focused on the prevention of HIV and HBV among migrants from Latin America [46–51,53,55–57,63] and Asian countries [40,42,43,45,52,54,58,61,62], respectively. HIV/STIs were mentioned only in four studies [47,53,55,64] and sexual health promotion in just two [39,65]. Migrants from sub-Saharan Africa were included only in four studies [39,64,66,67], despite being overrepresented among reported HIV cases in most receiving countries and thus a priority group or key population for prevention efforts. Moreover, migratory status was defined in different ways (country of birth/citizenship, ethnicity, reasons for migration) and several terms were used to identify the study population in the included papers. This confirms the lack of internationally accepted definitions to identify people who leave their countries to live/settle in other countries. The wide use of all-encompassing terms such as migrants, immigrants, ethnic origin and citizenship disregards the heterogeneity of this group making it difficult to focus on specific subgroups with increased vulnerability. For instance, vulnerable subgroups, such as migrant youth [39], men who have sex with men [51,57], transgender people, sex workers [59], people living with HIV [67], asylum seekers and undocumented migrants were rarely targeted in the included studies. The vulnerability of refugees, asylum seekers and undocumented women was highlighted in a study conducted in Belgium and the Netherlands that showed that most of them were extremely vulnerable to violence including sexual violence such as rape and sexual exploitation, which involve an increased risk for HIV/STIs [70].

### Improvement in knowledge, attitudes and intentions, but reported effects on testing and sexual risk behaviours were inconsistent

This review further shows that intervention effects vary across studies and measured outcomes. While effects on knowledge, attitudes, perceptions, behavioural intentions and skills were largely positive, the reported effects on testing and sexual risk behaviours were inconsistent and varied across studies from positive to negative, limited or no effect. This suggests that changing people’s (sexual) behaviours is challenging, and that knowledge and information explain only a small part of behaviour as there might indeed be other factors that play a crucial role in determining these behaviours [71]. Other studies have suggested that the fear of stigma and legal consequences of a positive HIV test may prevent migrants from being tested [15,72]. Moreover, while testing is voluntary and free, access to treatment for certain migrant subgroups may be limited in some countries [73]. Despite these factors, this review shows a lack of studies that evaluated structural factors that can limit migrants’ access to treatment and prevention services and thus increase their vulnerability.

### Prevention strategies incorporating structural interventions are needed

It is evident that most interventions in this review were behavioural [39,41,43,44,46,48–50,55,59–61,63–66] and focused on changing knowledge, perceptions, behaviour and attitudes through exposure to media campaigns, information or education programmes. In some studies, the behavioural interventions also included biomedical outcomes, such as HIV [51,53,56,57] and HBV testing [40,52,62] and HBV vaccination [58] or chlamydia incidence [47]. Only four of the studies included interventions that exclusively evaluated biomedical outcomes [42,45,54,67] and another just two/four evaluated traditional views on gender roles [46], IPV [47] and HIV related stigma that could be considered as structural indicators. However, none of the included studies addressed structural factors such as policies and laws that could limit migrants’ access to available services. Laws, policies and practices, as well as cultural and societal norms, shape the socio-environmental context in which HIV risk and preventive practices are generated [17] and can thus jeopardise preventive work and access on equal terms. Aung et al., (2017) underlined in their systematic review the need for interventions that also address the broader health system and structural factors that contribute to late HIV diagnoses in vulnerable subgroups of migrant populations and thus further transmission and poorer health outcomes [35]. However, the issue is not to replace behavioural and biomedical interventions with structural interventions but rather to incorporate them into a comprehensive, multilevel and multisectoral response [17].

### The right perspective without specifically integrating SRHR

The review further reveals that almost all interventions in the included studies aimed at improving availability, accessibility, acceptability and quality of HIV/STIs prevention programs targeting migrants suggesting thereby the application of the UN’s AAA-Q concept on the right to health [20]. Thus, none of the interventions specifically integrated a SRHR perspective that goes beyond a disease specific to a holistic perspective that recognizes the inherent connections between SRHR and HIV and the necessity of a bi-directional integration of SRHR and HIV services [19]. The starting point for most interventions in the included papers is that migration increases vulnerability towards HIV/STIs and that HIV-related care and preventative work needs to be responsive to the needs of migrants in order to improve access (32). This involved using outreach activities, mother tongues and peer educators or key persons from target groups. McMahon & Ward (2012), in their ‘realist review’ of evidence to guide targeted approaches to behavioural HIV prevention aimed at migrants in host countries, also found that incorporating cultural values into the intervention content and using appropriate languages (‘mother tongue’) and settings are critical elements in culturally appropriate HIV prevention programmes [28]. Although the UN’s AAA-Q concept is in line with a rights perspective, it does not systematically involve the integration of an SRHR perspective into the preventive work to address pregnancy, gender-based violence and inequality issues [19]. For example, condom use was only promoted for the prevention of HIV/STIs, but not for unintended pregnancies. Moreover, intimate partner violence was addressed as a risk factor for HIV/STI transmission, not as a violation of rights [47]. It is more about fulfilling the individual right to access services and be protected from HIV/STIs without a full scope of SRHR needs. HIV and SRHR linkages may not only prevent the spread of HIV/STIs, but also promote SRHR, reduce HIV-related stigma and discrimination and increase access to and utilisation of both HIV and SRHR services for vulnerable and marginalised subgroups of migrants, including transgender people, injecting drug users, sex workers, migrant youth and people living with HIV/AIDS. Future research in this field should therefore take this into consideration.

## Limitations

The aim of a scoping review is to map a research area, summarise research results and identify knowledge gaps in the existing literature [31]. Nevertheless, it is vital to evaluate the evidence. One of the limitations of this review is that most of the included studies were graded as medium or low-quality studies according to the checklist in the ‘TREND statement’ [37]. Several studies in the included papers lack information on theories, randomisation procedures, sample size calculations and the validation of measurement instruments. Some of the studies have large dropouts and there is no information on how dropouts and missing data were handled. Outcome measures in most of studies were self-reported and it is unclear in some studies whether blinding was applied. In a number of studies, measurements took place immediately after the intervention or during a short follow-up. There are also issues about the generalisability of the results in several studies due to the use of a non-random sampling strategy and there exists a lack of information about whether the study populations were similar to the target groups. Moreover, in several studies that included theory-based interventions, the results were not interpreted accordingly.

Another limitation is that the inclusion and exclusion criteria, as well as the definition of the term migrant used in the review, have led to the exclusion of some studies where the proportion of migrants (foreign-born) was not specified or because they represented less than 50% of the sample. The focus in these studies was on ethnicity rather than migration. For the same reason, no study on African migrants to the USA or the UK has been included because they were referred to as African Americans and ‘Black Africans’, respectively, without data on the country of birth or whether they were foreign-born. It is therefore possible that some literature of relevance to the research question has not been included.

## Conclusion

Existing research on HIV/STI prevention targeting migrants is limited and dominated by moderate to low-quality studies from the USA that lacked an SRHR perspective. These studies largely evaluated HIV and HBV behavioural interventions targeting Hispanic and Asian migrants, respectively, and where the UN’s AAAQ concept on the right to health is applied without specifically integrating an SRHR perspective. There is a need for good quality research, particularly in parts of the world other than the USA that will address all STIs and specifically target the most vulnerable subgroups of migrants. Further research requires greater scope and depth, including the need to apply an SRHR perspective, and incorporate biomedical and structural interventions to address the interacting causes of migrants’ vulnerability to HIV/STIs.

## Supplementary Material

Supplemental Material

## Paper context

HIV disproportionately affects migrants in Sweden. This scoping review was commissioned by the Public Health Agency of Sweden. Existing research is limited and dominated by HIV and HBV behavioural intervention studies from the USA that lacked a SRHR perspective. More research is needed, particularly outside the USA. Such research should tackle all STIs, specifically target vulnerable migrants, adopt an SRHR perspective and incorporate biomedical and structural interventions to address the interacting causes of migrants’ vulnerability.
