# Supplementary material for: Evidence and gaps in the literature on HIV/STI prevention interventions targeting migrants in receiving countries: a scoping review
Source: Glob Health Action. 2021 Aug 18;14(1):1962039. doi: 10.1080/16549716.2021.1962039 (PMC8381899; doi:10.1080/16549716.2021.1962039)
Supplement: Supplemental Material [file ZGHA_A_1962039_SM2276.zip › Supplementary files/Appendix 1.docx]

**Appendix 1. Example of search for literature in CINHAL database_1_**

| Search ID# | Search Terms | Search Options | Last run Via | Results |
| --- | --- | --- | --- | --- |
| S3 | ( Migrants OR Immigrants OR Transients OR Refugees OR Asylum Seekers ) AND ( Human Immunodeficiency Virus OR Acquired Immunodeficiency Syndrome OR Sexually Transmitted Diseases OR Sexually Transmitted Infections OR Sexual Health OR Sexuality ) AND ( Effectiveness OR Sexual Violence OR Gender Violence OR Incidence OR Prevalence OR Sexual Partners OR Unprotected Sex OR Unwanted Pregnancy OR Abortion OR Availability OR Accessibility OR Acceptability OR Adaptability OR Knowledge OR Attitude OR Behaviour OR Practices OR Stigma OR Discrimination OR Self Efficacy OR Policy OR Cultural Responsiveness ) AND ( Experimental Designs OR Longitudinal Study OR Random Assignment OR Randomized Trials OR Control Group OR Evaluation ) | **Expanders** - Apply related words **Search modes** - Find all my search terms | **Interface** - EBSCOhost Research Databases **Search Screen** - Advanced Search **Database** - CINAHL with Full Text | 562 |
| S2 | ( Migrants OR Immigrants OR Transients OR Refugees OR Asylum Seekers ) AND ( Human Immunodeficiency Virus OR Acquired Immunodeficiency Syndrome OR Sexually Transmitted Diseases OR Sexually Transmitted Infections OR Sexual Health OR Sexuality ) AND ( Effectiveness OR Sexual Violence OR Gender Violence OR Incidence OR Prevalence OR Sexual Partners OR Unprotected Sex OR Unwanted Pregnancy OR Abortion OR Availability OR Accessibility OR Acceptability OR Adaptability OR Knowledge OR Attitude OR Behaviour OR Practices OR Stigma OR Discrimination OR Self Efficacy OR Policy OR Cultural Responsiveness ) | **Expanders** - Apply related words **Search modes** - Find all my search terms | **Interface** - EBSCOhost Research Databases **Search Screen** - Advanced Search **Database** - CINAHL with Full Text | 1,903 |
| S1 | (Migrants OR Immigrants OR Transients OR Refugees OR Asylum Seekers) AND (Human Immunodeficiency Virus OR Acquired Immunodeficiency Syndrome OR Sexually Transmitted Diseases OR Sexually Transmitted Infections OR Sexual Health OR Sexuality) | **Expanders** - Apply related words **Search modes** - Find all my search terms | **Interface** - EBSCOhost Research Databases **Search Screen** - Advanced Search **Database** - CINAHL with Full Text | 2,591 |

_1_Literature search performed: June 13, 2019
