# Supplementary material for: Evidence and gaps in the literature on HIV/STI prevention interventions targeting migrants in receiving countries: a scoping review
Source: Glob Health Action. 2021 Aug 18;14(1):1962039. doi: 10.1080/16549716.2021.1962039 (PMC8381899; doi:10.1080/16549716.2021.1962039)
Supplement: Supplemental Material [file ZGHA_A_1962039_SM2276.zip › Supplementary files/Appendix 2.docx]

**Appendix 2. Charting Forms**

1. Study characteristics

| Publication (Record) details | Study details | Quality Assessment (TREND Statement) |
| --- | --- | --- |
| Authors, Year: Title  *Journal Volume (Issue number). doi:* | **AIM**:  **THEORY**:  **METHODS**  **Design**:  **Eligibility:**  **Recruitment and sampling Method:**  **Recruitment sites**:  **Settings**:  **Intervention**:  **Study period:**  **Sample size details:**  **Assignment method:**  **Sample**:  **Sample profile: *Sex****,* ***Age****,* ***Proportion of migrants****,* ***Country of origin****,* ***Length of stay***  **Follow up:**  **Retention rate**:  **Comparison**:  **Blinding**:  **Instrument**:  **Outcome measures:**  **Statistical analysis**:  **RESULTS**  **Baseline equivalence**: Unclear  **Outcomes and Estimation**:  **DISCUSSION**  **Interpretation**:  **Generalizability (External validity)** | **TREND Score:** (max=22)  **Limitations (TREND):**  **Other Limitations:**  **Generalizability (limited):**  **Overall quality**: |

1. Intervention characteristics

| Authors, year, “intervention n*ame”/publication title* and location | Intervention description | Intervention effects:  Improve, positive or increase:  Same or similar:  Decrease or negative or limited: |
| --- | --- | --- |
|  | **Theory**:  **Topic**:  **Targeted group**:  **Content**:  **Delivery method**:  **Unit of delivery**:  **Deliverer**:  **Setting**:  **Number and duration of sessions**:  **Time span**:  **Language**:  **Incentive**: |  |
