# Supplementary material for: Evidence and gaps in the literature on HIV/STI prevention interventions targeting migrants in receiving countries: a scoping review
Source: Glob Health Action. 2021 Aug 18;14(1):1962039. doi: 10.1080/16549716.2021.1962039 (PMC8381899; doi:10.1080/16549716.2021.1962039)
Supplement: Supplemental Material [file ZGHA_A_1962039_SM2276.zip › Supplementary files/supplementary.docx]

**Supplemental online material**

Appendix 1: Example of search for literature in CINHAL database (June13, 2019)

Appendix 2: Charting Forms
